# Predictors of Glycemic Response to Sulfonylurea Therapy in Type 2 Diabetes Over 12 Months: Comparative Analysis of Linear Regression and Machine Learning Models

**DOI:** 10.2196/82635

**Published:** 2026-02-06

**Authors:** Shilpa Garg, Robert Kitchen, Ramneek Gupta, Emanuele Trucco, Ewan Pearson

**Affiliations:** 1 Diabetes Endocrinology and Reproductive Biology, School of Medicine, University of Dundee, Nethergate, Dundee, Dundee, DD14HN, United Kingdom, 44 7443787733; 2 Novo Nordisk, Oxford, United Kingdom; 3 Disease Intelligence Pte Ltd, 10 Anson RoadSingapore, 79903, Singapore; 4 Computing Department, School of Science and Engineering, University of Dundee, Dundee, United Kingdom

**Keywords:** drug response, glycated hemoglobin, linear regression, machine learning models, treatment response prediction, type 2 diabetes

## Abstract

**Background:**

Sulfonylureas are commonly prescribed for managing type 2 diabetes, yet treatment responses vary significantly among individuals. Although advances in machine learning (ML) may enhance predictive capabilities compared to traditional statistical methods, their practical utility in real-world clinical environments remains uncertain.

**Objective:**

This study aimed to evaluate and compare the predictive performance of linear regression models with several ML approaches for predicting glycemic response to sulfonylurea therapy using routine clinical data, and to assess model interpretability using Shapley Additive Explanations (SHAP) analysis as a secondary analysis.

**Methods:**

A cohort of 7557 individuals with type 2 diabetes who initiated sulfonylurea therapy was analyzed, with all patients followed for 1 year. Linear and logistic regression models were used as baseline comparisons. A range of ML models was trained to predict the continuous change in hemoglobin A_1c_ (HbA_1c_) levels and the achievement of HbA_1c_ <58 mmol/mol at follow-up. These models included random forest, extreme gradient boosting, support vector machines, a conventional feedforward neural network, and Bayesian additive regression trees. Model performance was assessed using standard metrics including *R*² and root mean squared error for regression tasks and area under the receiver operating characteristic for classification. In a subset of 2361 patients, nonfasting connecting peptide (C-peptide) was analyzed as a proxy for β-cell function. SHAP analysis was performed to identify and compare key predictors driving model performance across methods.

**Results:**

All models exhibited similar performance, with no significant advantages of ML techniques over linear regression. For continuous outcomes, Bayesian additive regression trees demonstrated the highest *R*² (0.445) and lowest root mean squared error (0.105), though the differences among models were minimal. For the binary outcome, extreme gradient boosting achieved the highest area under the receiver operating characteristic curve (0.712), with CIs overlapping those of other models. Across all models, baseline HbA_1c_ was consistently the primary predictor, explaining the majority of the variance. SHAP analyses confirmed that baseline HbA_1c_, age, BMI, and sex were the most influential predictors. Sensitivity analyses and hyperparameter tuning did not significantly improve model performance. In the C-peptide subset, higher C-peptide levels were associated with greater glycemic improvement (*β*=−3.2 mmol/mol per log(C-peptide); *P*<.001).

**Conclusions:**

In this large, population-based cohort, ML models did not outperform traditional regression for predicting glycemic response to sulfonylureas. These findings suggest that limited gains from ML likely reflect an absence of strong nonlinear or high-order interactions in routine clinical data and that available features may not capture sufficient biological heterogeneity for complex models to confer added benefit. The inclusion of a C-peptide subset provides additional mechanistic insight by linking preserved β-cell function with treatment response.

## Introduction

Sulfonylureas are among the most commonly prescribed classes of glucose-lowering medications for individuals with type 2 diabetes. Their cost-effectiveness and accessibility make them particularly valuable in resource-constrained settings [1]. However, significant variability exists in glycemic responses among individuals. This variability is influenced by various clinical and biological factors, such as age, kidney function, and genetic predispositions [2,3]. Identifying predictors of treatment response is essential for advancing precision medicine approaches and minimizing trial-and-error prescribing practices [4].

Because sulfonylureas lower glucose primarily by stimulating insulin secretion from pancreatic β-cells, the degree of preserved β-cell function, often estimated by circulating connecting peptide (C-peptide) [5], may influence treatment response. However, such markers are rarely available in real-world datasets and are not routinely included in prediction studies.

Machine learning (ML) methods have shown promise in predicting treatment responses more accurately than traditional regression models, particularly due to their ability to handle complex, nonlinear interactions between variables without requiring prespecified assumptions [6,7]. In this context, ML approaches can capture subtle, multidimensional relationships that may be overlooked by traditional models, efficiently process large-scale longitudinal data, and generate data-driven insights that inform treatment selection. ML also offers better integration of diverse data types and improved interpretability through explainable AI, increasing clinical applicability [8]. Despite this promise, relatively few studies have focused on modeling glycemic response in diabetes using real-world data [9]. This gap in research presents a significant opportunity for further investigation.

Here, we use sulfonylurea response as an exemplar of diabetes drug response, due to its widespread use and the availability of clinical data. We evaluate and compare the efficacy of 5 ML models, including random forest, support vector machines, extreme gradient boosting (XGBoost), a conventional feedforward neural network (NN), and Bayesian additive regression trees (BART), in predicting the glycemic response to sulfonylureas in patients with type 2 diabetes. These models are compared with standard linear and logistic regression for continuous (change in hemoglobin A_1c_ [HbA_1c_]) and binary (achievement of HbA_1c_ <58 mmol/mol) outcomes. Analyses were conducted using a large real-world cohort from the GoDARTS (Genetics of Diabetes Audit and Research in Tayside Scotland) study, including a biologically informative subset with C-peptide measurements to assess the contribution of β-cell function.

In addition to comparing predictive performance across models, we conducted a secondary analysis to examine feature contributions using Shapley Additive Explanations (SHAP) [10]. This analysis allowed us to determine whether ML-derived feature importance aligns with the predictors identified by traditional regression approaches, providing insight into clinical interpretability and the practical utility of ML for informing treatment choice.

## Methods

### Study Population

The data were obtained from the GoDARTS [11]. This population-based cohort links prescription, clinical, and laboratory records for individuals with diabetes in Tayside and Fife. The inclusion criteria included patients with type 2 diabetes who initiated sulfonylurea therapy (either as monotherapy or in combination), had a baseline HbA_1c_ measurement (defined as the closest value within 183 days before to 7 days after treatment initiation), and a follow-up HbA_1c_ measurement after a 1-year period. The 183-day window was selected to balance data availability and clinical relevance. For this analysis, only 2 HbA_1c_ values per patient were used, 1 at baseline and 1 at follow-up, in line with the model’s aim of predicting glycemic response from initial clinical features.

### Ethical Considerations

Data provision and linkage were carried out by the University of Dundee Health Informatics Centre, with analysis of anonymized data performed in an ISO27001 and Scottish Government–accredited secure safe haven. Health Informatics Centre standard operating procedures were reviewed and approved by the National Health Service (NHS) East of Scotland Research Ethics Service (22/ES/0034), and consent for this study was obtained from the NHS Fife Caldicott Guardian. Under these approvals, secondary analysis of anonymised routine healthcare data does not require additional participant consent or compensation.

### Baseline Predictor Variables

Baseline clinical features included age, sex, HbA_1c_, BMI, total cholesterol, high-density lipoprotein (HDL) cholesterol, smoking status, systolic blood pressure, alkaline phosphatase, alanine transaminase, serum potassium, serum creatinine, bilirubin, and albumin. These variables were selected based on their availability in routine care and their known or suspected relevance to glycemic outcomes [12]. Except for baseline HbA_1c_ (as defined above), all measurements were defined as the closest recorded value within 2 years before to 90 days after sulfonylurea initiation.

To estimate average daily sulfonylurea dose, prescription records were used to extract drug strength and quantity dispensed. Five sulfonylureas were included: gliclazide, glipizide, glimepiride, glibenclamide, and tolbutamide. Each prescription’s dose was standardized by dividing the prescribed dose by the drug’s maximum recommended daily dose (as per the British National Formulary). This yielded a standardized dose unit, which was then multiplied by the number of tablets prescribed per prescription to calculate the total standardized dose. For each patient, the total dose was summed across all prescriptions, excluding the last one, and divided by treatment duration to derive the average daily dose. This dose was then categorized into low, medium, and high using quartiles.

### Outcome Definitions

The primary continuous outcome was defined as the change in glycated hemoglobin (HbA_1c_), measured in millimoles per mole, from baseline (at the time of sulfonylurea initiation) to the follow-up measurement closest to 12 months, within a window of 6-15 months.

The binary outcome was defined as whether a patient achieved a follow-up HbA_1c_ level below 58 mmol/mol.

### Data Preparation

To ensure consistency and compatibility with ML models, several preprocessing steps were applied. Continuous variables with skewed distributions underwent log transformation to approximate a normal distribution [13], enhancing model stability and reducing the influence of extreme values. Following this, all continuous predictors, including laboratory test results and physiological measurements, were scaled to a range between 0 and 1 using min-max normalization [14]. This rescaling placed variables on a uniform scale, which is particularly important for algorithms like NNs that are sensitive to variable magnitudes. Categorical variables (eg, sex, smoking status, treatment group, average daily dose) were converted using one-hot encoding to make them compatible with model inputs.

### Missing Data Imputation and Collinearity Assessment

Patients missing either baseline or follow-up HbA_1c_ measurements were excluded. For remaining clinical predictors, missingness was below 10% and not clustered within specific individuals. Missing values were imputed using multiple imputation by chained equations [15] implemented in R (mice v3.18.0). Five imputed datasets were generated with 50 iterations each, using predictive mean matching for continuous variables. Full details of the imputation model are provided in Section 1 in Multimedia Appendix 1. Convergence was assessed using the mean and variance of each variable across iterations and comparing distributions of observed and imputed values. Analyses were performed on pooled estimates derived using Rubin’s rules [16].

Collinearity among predictors was evaluated using variance inflation factors (VIFs) [17]. Predictors with VIF values greater than 5 were reviewed for redundancy. In our final models, VIFs ranged from 1.06 to 1.5, indicating no meaningful multicollinearity. As a sensitivity check, strongly correlated clinical variables (*r* > 0.8) were examined, and when overlap occurred (eg, estimated glomerular filtration rate vs serum creatinine), the variable more routinely and reliably measured in clinical practice (serum creatinine) was retained.

### Statistical Analysis: Baseline Models

Initial statistical analyses were conducted using linear regression [18] for the continuous outcome and logistic regression for the binary outcome. These models identified baseline associations between clinical predictors and glycemic response to sulfonylurea therapy. Logistic regression estimated the probability of achieving an HbA_1c_ <58 mmol/mol. Of the 7557 individuals included, 3818 achieved the target, and 3739 did not.

### Residualization of Baseline HbA_1c_

To disentangle treatment response from baseline glycemia, change in HbA_1c_ was regressed on baseline HbA_1c_. The residuals from this model were used as outcomes for ML analyses. This allowed the identification of predictors influencing glycemic response independent of baseline HbA_1c_ levels [19].

### ML Models

Five ML models were implemented, reflecting diverse algorithmic strategies:

1. Random forest: An ensemble method that constructs multiple decision trees on bootstrapped data and aggregates their predictions [20].
2. Support vector machines: A kernel-based classifier that establishes an optimal separating hyperplane [21].
3. XGBoost: A boosting technique that sequentially builds trees to minimize residual error [22].
4. NNs: A conventional feedforward NN (multilayer perceptron) trained using resilient backpropagation. Comprising layers of interconnected neurons, NNs excel at modeling complex relationships and require larger datasets and regularization to mitigate overfitting [23].
5. BARTs: A Bayesian ensemble method that combines multiple regression trees and estimates uncertainty in predictions [24]. BART is noted for strong performance in clinical applications [25-27].

### Model Implementation

For model development, a 2-stage validation framework combining cross-validation and a held-out test set was used. The data were randomly split into a 70% training set and a 30% held-out test set. Within the training set, 10-fold cross-validation [18] was used for hyperparameter tuning and model selection to enhance model stability and reduce overfitting. Final performance was evaluated on the held-out test set, which remained unseen during training.

All analyses were performed in R (version 4.3.0). A detailed description of the packages and functions used for each model is presented in Section 2 in Multimedia Appendix 1, and XGBoost and NN hyperparameters are provided in Sections 3 and 4 in Multimedia Appendix 1, respectively. All preprocessing and modeling code is publicly available on GitHub [28].

### Feature Importance

To identify the clinical features most strongly influencing model predictions, we assessed feature importance using SHAP values along with the built-in variable importance metrics from each model. SHAP values quantify the contribution of individual predictors to model outputs, enabling transparent, model-agnostic interpretation. Although SHAP can be applied to multiple model types, our results focused on the XGBoost model because it showed optimal predictive performance. SHAP summary plots were generated to visualize the magnitude and direction of feature effects, ranking predictors by their mean absolute SHAP values. Comparative plots across models were generated to visualize predictor impact on treatment response. This unified approach supported consistent evaluation of feature relevance across models and enhanced clinical interpretability.

### Performance Evaluation

Model performance was assessed separately for the continuous and binary outcomes. For the continuous outcome, evaluation metrics included root mean squared error (RMSE), mean absolute error, and the coefficient of determination (*R*²), which indicates the proportion of variance in the outcome explained by the model [29,30].

For the binary outcome, performance was evaluated using standard classification metrics: area under the receiver operating characteristic curve (AUC), accuracy, sensitivity (recall), and specificity [31,32]. In the linear regression models, regression coefficients were interpreted to assess the direction and magnitude of each predictor’s association with the outcome, while *P* values indicated the statistical significance of these associations. An *R*² value provided an overall measure of model fit, and a *P* value below .05 was considered statistically significant.

To evaluate differences in performance across models, a resampling-based approach was used to compare their predictive metrics [33]. Pairwise comparisons were conducted to assess whether any model significantly outperformed the others.

## Results

### Cohort Characteristics

The study included 7557 individuals with type 2 diabetes who initiated sulfonylurea therapy and had both baseline and follow-up HbA_1c_ values available. The cohort had a mean age of 63.7 (SD 11.8) years, and 57.9% (n=4377) were male. The mean baseline HbA_1c_ was 76.5 (SD 16.7) mmol/mol (Table 1).

**Table 1.** Baseline demographic and clinical characteristics of the study population.

Clinical variable	Sulfonylurea cohort (N=7557)
Age at therapy initiation (y), mean (SD)	63.7 (11.8)
Sex, n (%)	
Male	4377 (57.9)
Female	3180 (42.1)
Average daily dose, n (%)	
Low	1844 (24.4)
Medium	3822 (50.6)
High	1891 (25)
Duration of diabetes (y), mean (SD)	4.96 (4.49)
Duration of diabetes, n (%)	
0‐1 years	1416 (18.7)
1‐5 years	3099 (41)
>5 years	3042 (40.3)
Time of treatment (mo), mean (SD)	11.4 (2.2)
Time from baseline HbA_1c_^a^ measurement to treatment start (d), mean (SD)	21.3 (29.1)
Year of drug start, mean (SD)	2010 (6.12)
BMI (kg/m^2^), mean (SD)	31.3 (6.3)
Total cholesterol (mmol/L), mean (SD)	4.5 (1.2)
HDL^b^ cholesterol (mmol/L), mean (SD)	1.2 (0.3)
Serum creatinine (µmol/L), mean (SD)	80.3 (27.4)
Albumin (g/L), mean (SD)	42.2 (4.0)
Bilirubin (µmol/L), mean (SD)	9.9 (5.2)
Alkaline phosphatase (U/L), mean (SD)	89.5 (42.1)
ALT/SGPT^c^ (U/L), mean (SD)	34.2 (24.4)
Potassium (mmol/L), mean (SD)	4.4 (0.4)
Systolic blood pressure, mean (SD)	137 (17.4)
Smoking status, n (%)	
Ever smoked—yes	5628 (74.5)
Ever smoked—no	1870 (24.7)
Ever smoked—unknown	59 (0.8)
Therapy group, n (%)	
Mono	2508 (33.2)
Dual	4251 (56.3)
Triple	798 (10.6)
Index of multiple deprivation quintile, n (%)	
1 (most deprived)	1583 (16.5)
2	1609 (21.3)
3	1497 (19.8)
4	1396 (18.5)
5 (least deprived)	1246 (16.5)
Unknown	226 (3.0)
Ethnicity, n (%)	
White	5696 (75.4)
Others/mixed	259 (3.4)
Missing	1602 (21.2)
Region, n (%)	
Tayside	5965 (78.9)
Fife	1592 (21.1)
Baseline HbA_1c_ (mmol/mol), mean (SD)	76.5 (16.7)
HbA_1c_ outcome (mmol/mol) (treatment HbA_1c_), mean (SD)	61.1 (15.4)
HbA_1c_ response (change from baseline; mmol/mol), mean (SD)	–15.4 (18)

a HbA_1c_: hemoglobin A_1c_.

b HDL: high-density lipoprotein.

c ALT/SGPT: alanine aminotransferase/serum glutamate pyruvate transaminase.

### Associations Between Clinical Covariates and Treatment Response

A linear regression model was fit to assess the relationship between clinical variables and the change in HbA_1c_, defined as the difference between baseline HbA_1c_ and follow-up values (ie, change=treatment–baseline HbA_1c_). A negative change in HbA_1c_ indicates a better treatment response to sulfonylureas, while a positive change signifies a worse response. No feature scaling (min-max normalization) was applied to the variables in this model. The model demonstrated satisfactory fit, with an *R*² value of 0.41 and a significant F-statistic (222.2; *P<*.001).

The linear regression analysis identified several clinical variables significantly associated with the change in HbA_1c_ among individuals treated with sulfonylureas. Older age and higher baseline HbA_1c_ were associated with greater HbA_1c_ reductions (negative coefficients), indicating more favorable responses. In contrast, a higher BMI was associated with smaller reductions, suggesting that individuals with higher BMI may struggle to achieve desired glycemic control. Additionally, sex differences indicated that male participants demonstrated slightly better response to sulfonylurea treatment than female participants.

**Figure 1.**
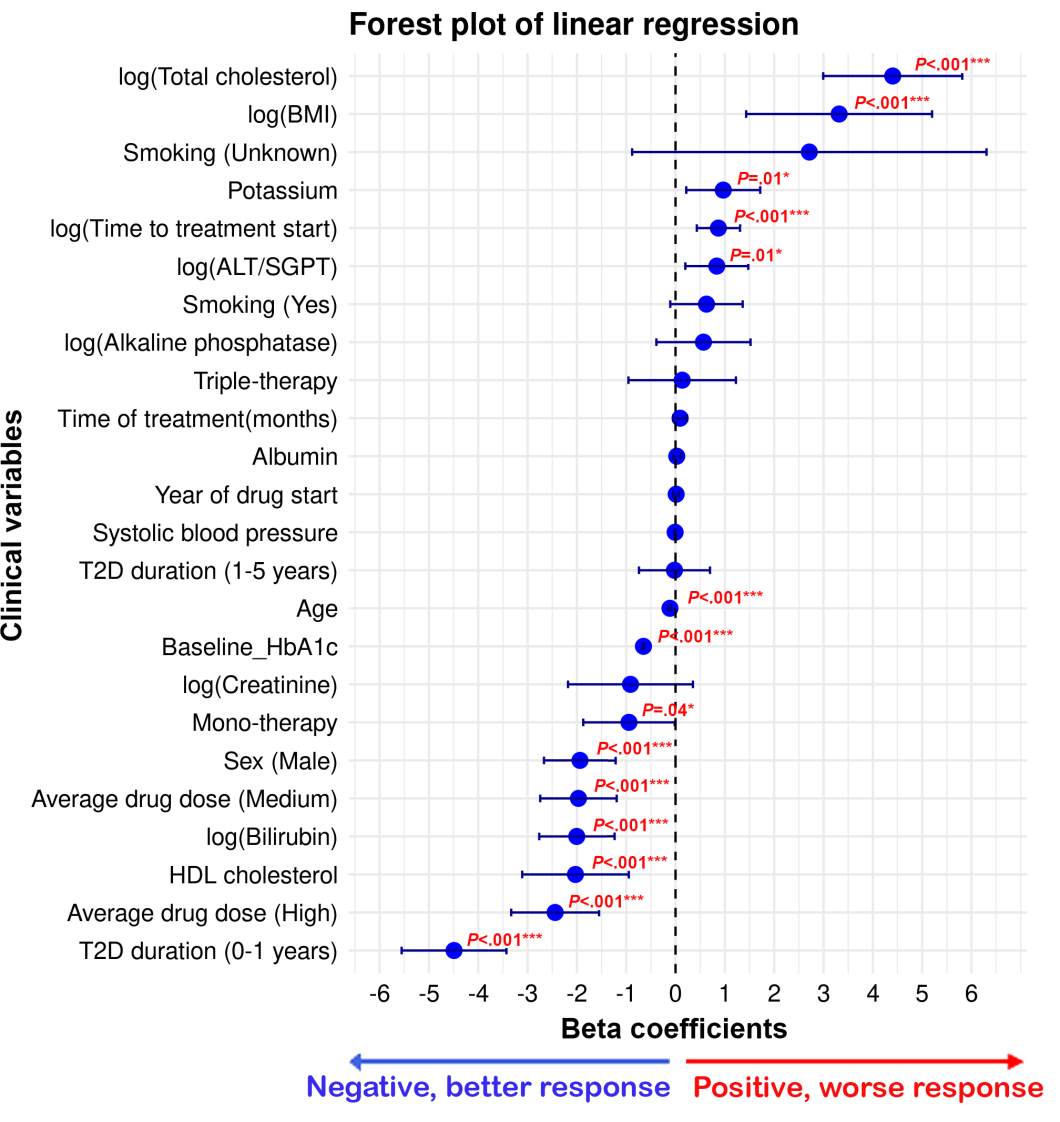
Forest plot showing regression coefficients and 95% CIs for predictors of change in hemoglobin A_1c_ (HbA_1c_). Points represent model estimates and horizontal lines indicate 95% CIs. ALT: alanine aminotransferase; SGPT: alanine aminotransferase/serum glutamate pyruvate transaminase; T2D: type 2 diabetes. *P<.05; **P<.01; ***P<.001.

These associations are illustrated in Figure 1, which presents a forest plot of the linear regression coefficients and CIs, highlighting the magnitude and direction of each predictor’s effect on HbA_1c_ change.

### C-Peptide Analysis

In a subset of 2361 individuals with nonfasting C-peptide data, higher C-peptide levels were strongly associated with greater reductions in HbA_1c_ at 12 months (linear regression: *β*=−3.2 mmol/mol per log(C-peptide); *P*<.001). This finding suggests that preserved endogenous insulin secretion contributes to more favorable treatment outcomes.

### ML Model Performance for Continuous Outcome

For the continuous outcome of predicting changes in HbA_1c_, several models were evaluated. The results indicate that the BART model exhibited the lowest RMSE of 0.105 (21%) and the lowest mean absolute error of 0.079 (16.1%), highlighting its effective performance in estimating continuous changes. XGBoost and NNs also performed comparably, with RMSE values of 0.106.

On the original HbA_1c_ scale, this corresponds to an approximate prediction error of 13.8 mmol/mol, comparable to the residual standard error from the linear regression model. Thus, the clinical prediction error was approximately ~14 mmol/mol.

However, the differences in RMSE and *R*^2^ across all models were minimal, indicating that while BART performed slightly better, the performance of all models was relatively comparable (Table 2). The similar *R*^2^ and RMSE values further suggest that no single model stands out significantly.

**Table 2.** Regression model performance metrics (root mean squared error [RMSE], mean absolute error [MAE], and *R*²) for continuous outcome prediction across all 6 models^a^.

Models	RMSE	MAE	*R* ^2^
Linear regression	0.106	0.08	0.434
RF^b^	0.108	0.082	0.424
SVM^c^	0.106	0.079	0.438
XGBoost^d^	0.106	0.08	0.433
NN^e^	0.106	0.081	0.427
BART^f^	0.105	0.079	0.445

a RMSE is shown as the normalized values.

b RF: random forest.

c SVM: support vector machine.

d XGBoost: extreme gradient boosting.

e NN: neural network.

f BART: Bayesian additive regression trees.

### Statistical Comparison of Model Performance

In addition to reporting standard performance metrics, statistical comparisons were performed using resampling-based techniques. Pairwise comparisons of RMSE and *R*² values across all models showed no statistically significant differences in performance; all ML models, including the linear regression baseline, performed similarly on this dataset.

### Sensitivity Analysis: Residualized HbA_1c_ Change

A sensitivity analysis was performed to evaluate predictors of HbA_1c_ change independent of baseline glycemia. Across all models, the maximum *R*^2^ value decreased to 0.05, indicating that only ~5% of the residual variance in 12-month HbA_1c_ response was explained by routine clinical features after removing the effect of baseline HbA_1c_.

The performance metrics from this analysis further indicated that the RMSE and *R*^2^ values remained consistent across most models (Table 3). However, XGBoost and BART showed poorer performance, with high RMSE and lower *R*^2^ values. This likely reflects the fact that these algorithms are better suited for large, high-dimensional, or highly nonlinear datasets, whereas the present dataset may not contain sufficient complexity. This consistency across most models suggests that while some approaches explain marginally more variance in the sensitivity analysis, their predictive accuracy in terms of mean squared error remains stable.

**Table 3.** Model performance after adjustment for baseline hemoglobin A_1c_ (HbA_1c_) across all 6 models.

Models	RMSE^a^	MAE^b^	*R* ^2^
Linear regression	0.127	0.095	0.054
RF^c^	0.126	0.095	0.056
SVM^d^	0.128	0.094	0.051
XGBoost^e^	0.230	0.183	0.01
NN^f^	0.127	0.095	0.053
BART^g^	0.214	0.191	0.021

a RMSE: root mean squared error.

b MAE: mean absolute error.

c RF: random forest.

d SVM: support vector machine.

e XGBoost: extreme gradient boosting.

f NN: neural network.

g BART: Bayesian additive regression trees.

### ML Model Performance for Binary Outcome

For the binary outcome of predicting achievement of HbA_1c_ <58 mmol/mol, model performance was assessed using the AUC and accuracy. The XGBoost model achieved the highest AUC (0.712), followed closely by BART (0.710), indicating modest discriminatory ability. Logistic regression performed similarly, with an AUC of 0.702.

The CIs for all models showed substantial overlap (ranging from 0.681 to 0.724 for logistic regression and 0.692 to 0.733 for XGBoost), indicating that no model demonstrated statistically superior discrimination. Overall, the models were broadly comparable in their ability to distinguish responders from nonresponders.

**Figure 2.**
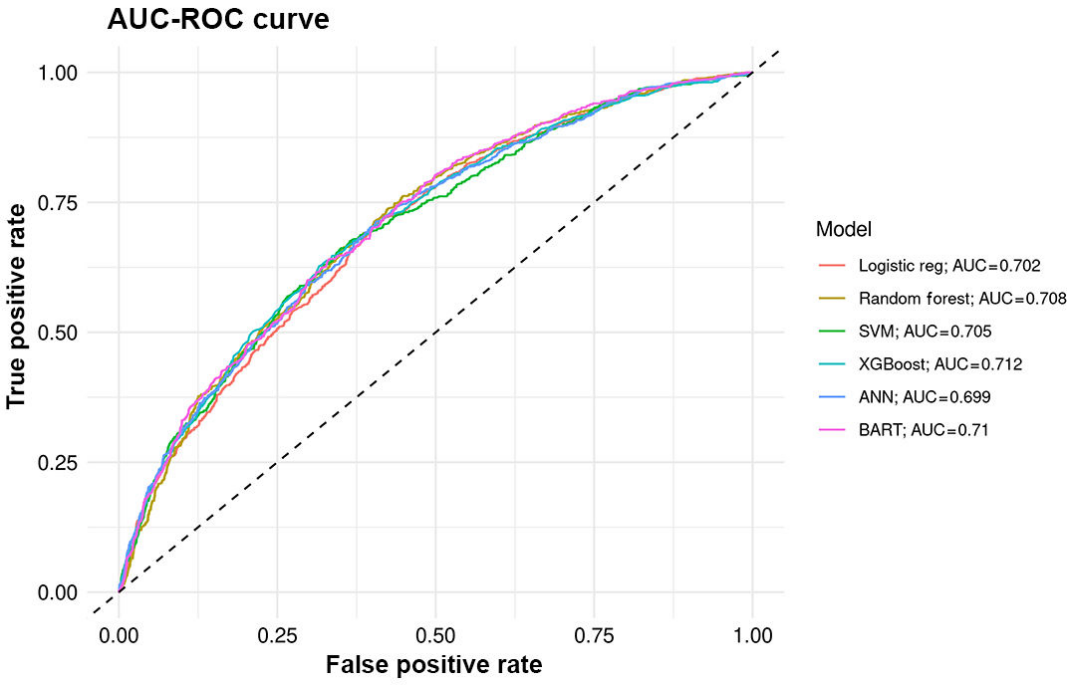
Receiver operating characteristic (ROC) curves for binary outcome prediction models. Colors correspond to individual models as shown in the legend. ANN: artificial neural network; AUC: area under the receiver operating characteristic curve; BART: Bayesian additive regression trees; SVM: support vector machine; XGBoost: extreme gradient boosting.

Model-level classification metrics are summarized in Table 4, and the corresponding ROC curves for all models are shown in Figure 2, illustrating their similar performance profiles. In Figure 2, colored curves represent the individual models, visually reinforcing the overlapping AUCs and the absence of meaningful differences in classification performance.

**Table 4.** Discrimination and classification performance of binary outcome models.

Models	AUC^a^ (95% CI)	Accuracy	Precision	Recall
Logistic regression	0.702 (0.681‐0.724)	0.654	0.657	0.628
RF^b^	0.708 (0.687‐0.729)	0.652	0.656	0.628
SVM^c^	0.705 (0.684‐0.727)	0.65	0.656	0.618
XGBoost^d^	0.712 (0.692‐0.733)	0.646	0.65	0.625
NN^e^	0.699 (0.678‐0.72)	0.645	0.645	0.636
BART^f^	0.71 (0.689‐0.731)	0.651	0.652	636

a AUC: area under the receiver operating characteristic curve.

b RF: random forest.

c SVM: support vector machine.

d XGBoost: extreme gradient boosting.

e NN: neural network.

f BART: Bayesian additive regression trees.

### Feature Importance and SHAP Interpretability

Feature importance analyses consistently identified baseline HbA_1c_ as the most significant predictor across all models. Other variables such as BMI, alanine transaminase, total cholesterol, and systolic blood pressure were also found to be significant, though their rankings varied slightly between algorithms. Across all 5 models, the rankings of key predictors remained largely consistent.

**Figure 3.**
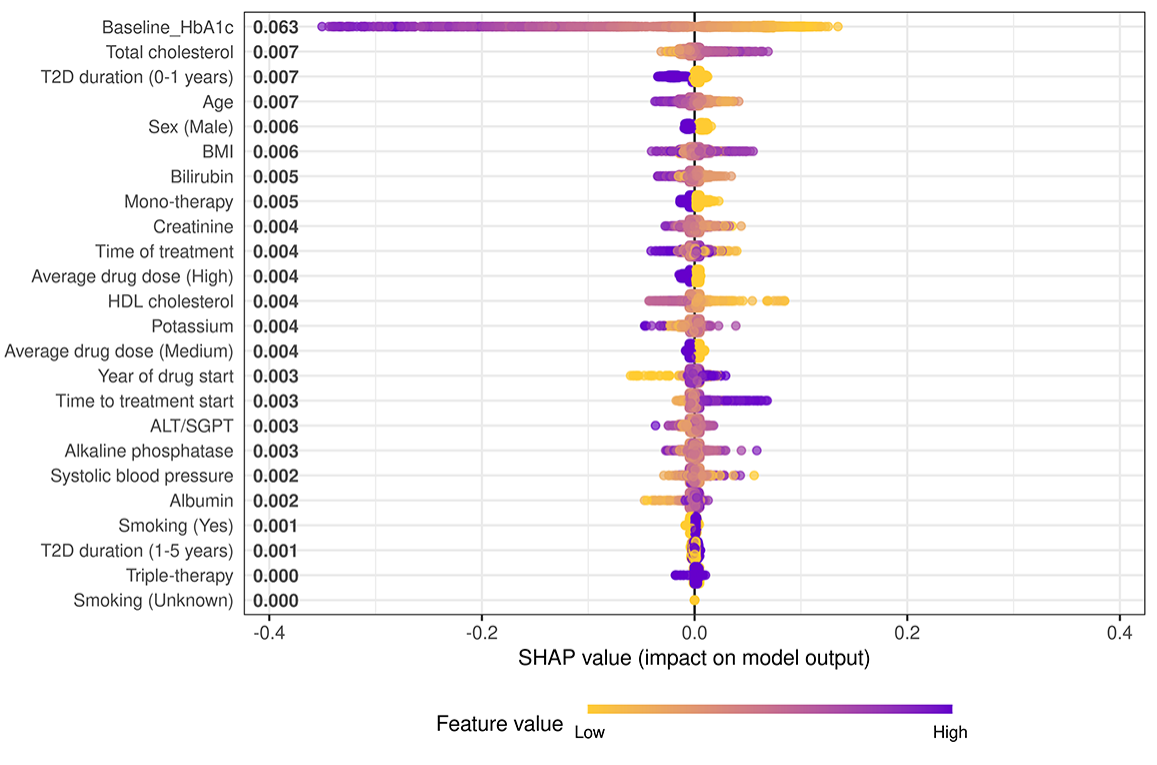
Shapley Additive Explanations (SHAP) summary plot of feature importance in predicting glycemic response. Each dot represents 1 patient. The x-axis indicates the SHAP value (impact on model output). The color gradient reflects feature values (blue=higher values; yellow=lower values). Features are ordered by mean absolute SHAP values, indicating overall contribution to model predictions. ALT/SGPT: alanine aminotransferase/serum glutamate pyruvate transaminase; HbA_1c_: hemoglobin A_1c_; HDL: high-density lipoprotein; T2D: type 2 diabetes.

To further explain feature contributions, a SHAP summary plot derived from the XGBoost model is presented (Figure 3), offering a more granular view of individual feature effects on model predictions. Baseline HbA_1c_ had the highest mean SHAP value (0.063), followed by total cholesterol, duration of diabetes, and age. Higher baseline HbA_1c_ values were associated with larger predicted reductions in HbA_1c_ (ie, more negative SHAP values), indicating greater expected treatment benefit.

The SHAP plot further shows that lower total cholesterol values corresponded to more negative SHAP values, suggesting better predicted outcomes, whereas higher cholesterol was linked to reduced response. Similarly, shorter diabetes duration and younger age were associated with more favorable predictions. For the variable sex, blue points represent male participants and yellow points represent female participants; male participants were associated with more negative SHAP values, indicating a better predicted response, compared to female participants, whose SHAP values clustered closer to or above zero.

## Discussion

### Principal Findings

This study compared the predictive performance of traditional regression models and a range of ML algorithms for predicting glycemic response to sulfonylurea therapy in individuals with type 2 diabetes. The primary finding is that, with the dataset used, all models demonstrated comparable predictive performance. No ML approach significantly outperformed standard regression for either the continuous outcome or the binary outcome. These results indicate that, within routinely collected clinical data, the additional algorithmic complexity of ML methods does not necessarily yield superior predictive accuracy. Regression therefore remains a robust and interpretable option for predicting drug response in this context.

Linear regression analysis revealed that the model explained approximately 43% of the variance in changes to HbA_1c_. In the sensitivity analysis, after adjusting for baseline HbA_1c_, the maximum *R*² across all models dropped to 0.05, indicating that only a small proportion of outcome variability was captured by the remaining routine clinical features. This highlights the need for additional or more informative biomarkers to improve prediction.

Additionally, only about 50% (n=3818) of the participants achieved glycemic control after 1 year of sulfonylurea therapy, despite the relatively homogeneous clinical characteristics of the cohort. This finding highlights considerable interindividual variability in treatment response, suggesting that additional biological and behavioral factors may shape drug efficacy. Such heterogeneity may reflect differences in pharmacodynamic sensitivity, medication adherence, β-cell reserve, and underlying insulin resistance. BMI and HDL were considered indirect proxies of insulin resistance, as higher BMI is typically associated with greater insulin resistance, whereas higher HDL levels are generally linked to improved insulin sensitivity. Consistent with this, participants with higher BMI had poorer glycemic response, while those with higher HDL tended to show more favorable outcomes. However, direct measures of insulin resistance were not available in this dataset.

### No Added Value From ML Methods

While multiple ML algorithms were evaluated in parallel with traditional regression models, none demonstrated superior predictive performance. Across both continuous and binary outcomes (Tables 2 and 4), differences in metrics such as RMSE, *R*², and AUC were small and not statistically significant, with overlapping CIs for all models. Even after hyperparameter tuning, predictive metrics remained modest, suggesting that ML methods did not uncover hidden patterns or interactions that traditional models missed.

This limited gain in predictive accuracy likely reflects the nature of routinely collected clinical data, which may lack sufficient biological complexity for ML algorithms to exploit. When input variables do not encompass detailed mechanistic information, even advanced algorithms cannot extract additional predictive signal. Consequently, transparent and easily interpretable models, such as linear or mixed-effects regression, may remain preferable, particularly when predictive performance is comparable. These models allow clinicians to understand feature contributions directly and translate findings into actionable treatment decisions.

Although complex ML models theoretically enable the capture of nonlinear relationships, their greater computational burden and reduced interpretability may limit their clinical utility unless they provide meaningful improvements in accuracy. The consistency of results across all modeling strategies, ranging from simple linear regression to ensemble and NN approaches, suggests that the available clinical features may not contain enough biological heterogeneity for ML methods to offer an advantage.

By intentionally comparing models of differing complexity, this study demonstrates that when data lack substantial nonlinearity or high-dimensional interactions, regression-based methods may remain more appropriate and efficient. This finding supports the continued reliance on interpretable models in routine clinical prediction tasks, where model parsimony and interpretability remain more valuable than algorithmic complexity for precision-medicine applications.

### Features That Inform Drug Response Prediction

Feature importance and SHAP analyses consistently identified baseline HbA_1c_, age, BMI, and sex as key predictors across regression and ML models. Baseline HbA_1c_ was the strongest predictor, reflecting both regression to the mean and true physiological responsiveness [34]. Older age and male sex were associated with greater HbA_1c_ reduction, whereas higher BMI predicted poorer response, aligning with evidence that adiposity may reduce sulfonylurea effectiveness [35]. These findings parallel results from the 5-drug predictive model developed by Dennis et al [36], suggesting that these core predictors generalize across therapeutic classes.

C-peptide, available for a subset of participants, showed a strong positive association with glycemic improvement, consistent with the insulin-secretagogue mechanism of sulfonylureas. This highlights the contribution of β-cell reserve to treatment heterogeneity and underscores the value of including mechanistic biomarkers to enhance model interpretability and predictive accuracy.

### Limitations

This study has several limitations. First, routinely collected clinical data omit key determinants of treatment response such as adherence, diet, physical activity, genetics, and social factors. The limited availability of C-peptide prevented fuller assessment of β-cell function, and its strong association with response suggests that the inclusion of mechanistic biomarkers would likely improve predictive accuracy.

Second, the study population was limited to patients from Tayside and Fife in Scotland, which may reduce the generalizability of the findings to other regions or health care systems with different population characteristics or clinical practices.

Third, treatment response was assessed using a single HbA_1c_ value taken between 6 and 15 months after treatment initiation. Although data closest to 12 months post-initiation were used, variability in the follow-up period (6‐15 mo) may introduce measurement variability and ought to be considered.

Additionally, the relatively low *R*² values across all models, even after applying rigorous methods such as a 70/30 train-test split and 10-fold cross-validation, suggest that the available clinical features alone do not explain sufficient variation in treatment response to support strong predictive performance.

### Future Directions

Future research should focus on improving prediction models by incorporating richer and more diverse data sources, including genetic, metabolomic, and continuous glucose monitoring data, as well as direct measures of insulin resistance and β-cell function. Integrating these modalities could improve model accuracy and help explain why individuals respond differently to sulfonylurea treatment. In addition, future studies could explore advanced modeling approaches, such as deep learning or hybrid models that balance predictive power with ease of interpretation for clinical use. The increasing availability of real-world, longitudinal clinical data also supports the use of time-dependent models, such as recurrent neural networks or transformer models, to track how treatment response evolves over time. Finally, testing these models in independent and ethnically diverse populations will be important to assess their generalizability and real-world applicability.

In conclusion, this study shows that the traditional regression models remain robust, clinically interpretable, and sufficient for predicting glycemic response to sulfonylurea therapy using routine data. The comparable performance of ML methods suggests that model transparency and accessibility may currently outweigh the small gains offered by algorithmic complexity in this context.

## Supplementary material

10.2196/82635 Multimedia Appendix 1 Missing data imputation.

## Abbreviations

AUC: area under the receiver operating characteristic curve
BART: Bayesian additive regression trees
C-peptide: connecting peptide
GoDARTS: Genetics of Diabetes Audit and Research in Tayside Scotland
HbA_1c_: hemoglobin A_1c_
HDL: high-density lipoprotein
ML: machine learning
NHS: National Health Service
NN: neural network
RMSE: root mean square error
SHAP: Shapley Additive Explanations
VIF: variance inflation factor
XGBoost: extreme gradient boosting
