# Supplementary material for: Predictors of Glycemic Response to Sulfonylurea Therapy in Type 2 Diabetes Over 12 Months: Comparative Analysis of Linear Regression and Machine Learning Models
Source: JMIR Diabetes. 2026 Feb 6;11:e82635. doi: 10.2196/82635 (PMC12880802; doi:10.2196/82635)
Supplement: Multimedia Appendix 1 [file diabetes-v11-e82635-s001.docx]

Supplementary Methods

## Section 1. Missing Data Imputation

Missing data were addressed using multivariate imputation by chained equations (MICE).

### 1.1 Variables Included in the Imputation Model

The following clinical and laboratory variables were included along with age and sex:

- Body Mass Index (BMI)
- Total cholesterol
- HDL cholesterol
- Systolic blood pressure
- Creatinine
- Potassium
- Albumin
- Alkaline phosphatase
- Bilirubin
- ALT (alanine aminotransferase)

### 1.2 Imputation Method

- Type of imputation: Predictive mean matching (PMM) for all continuous variables.
- Number of imputations: *m* = 5.
- Iterations: 50 cycles per imputation.

### 1.3 Diagnostics and Pooling

- Convergence and quality of imputations were evaluated using comparisons of means, variances, and empirical distributions across imputed datasets.
- Final parameter estimates were combined using Rubin’s rules.

## Section 2. Model Implementation

All predictive modelling was conducted in R (version 4.3.0). The following packages and functions were used for model development, training, cross-validation, feature importance estimation, and performance evaluation.

### 2.1 Regression and Classification Models

- Linear and Logistic Regression: Implemented using the base R glm() function.
- Random Forest (RF): Implemented using the randomForest package.
- Gradient Boosting (XGBoost): Implemented using the xgboost package. Feature attributions were obtained using the shap package, which provides SHAP values for tree-based models.
- Support Vector Machine (SVM): Implemented using the svm() function in the e1071 package.
- Artificial Neural Networks (ANN): Implemented using the neuralnet package.
- Bayesian Additive Regression Trees (BART): Implemented via the wbart() function from the BART package.

### 2.2 Workflow, Tuning, and Evaluation

- Cross-validation and model training: Conducted using the caret package, which provided standardized workflows for hyperparameter tuning and resampling.
- Performance metrics: Receiver operating characteristic (ROC) curves and area under the curve (AUC) values were obtained using the pROC package.
- Feature importance: Extracted using the varImp() function.
- Model comparison: Performed using resamples() from the caret package.
- Missing data imputation: Conducted using the MICE package (mice() and complete() functions).

## Section 3. XGBoost Implementation

### 3.1 Model Specification

- Regression models: objective = "reg:squarederror", booster = "gbtree"
- Classification models: objective = “binary:logistic”

Hyperparameters were tuned using grid search with 10-fold cross-validation.

### 3.2 Hyperparameter Grid Search

A grid of 108 unique hyperparameter combinations was evaluated:

- max_depth: 3, 5, 7, 9
- subsample: 0.5, 0.7, 1.0
- colsample_bytree: 0.5, 0.7, 1.0
- eta (learning rate): 0.01, 0.1, 0.2

Cross-validation was performed using xgb.cv(), with early stopping enabled to determine the optimal number of boosting rounds (nrounds).

### 3.3 Final Selected Hyperparameters

The best-performing classifier used the following parameters:

- max_depth: 3
- min_child_weight: 1
- subsample: 0.7
- colsample_bytree: 0.7
- eta: 0.1
- nrounds: 200

## Section 4. Artificial Neural Network Implementation

Neural network models were implemented using the neuralnet package.

### 4.1 Architecture

- Hidden layers: One hidden layer.
- Number of neurons:
  - Regression models: 2 neurons
  - Classification models: 3 neurons

### 4.2 Activation Function

- Rectified Linear Unit (ReLU) activation (act.fct = relu).

### 4.3 Optimization Strategy

- Optimizer: Resilient backpropagation (Rprop+), an adaptive gradient-based optimization method.
- Learning rate: Rprop does not use a conventional global learning rate; instead, it applies weight-specific adaptive update values (initial value = 0.1).

### 4.4 Training Specifications

- Batch size: Full-batch training (all observations per update).
- Epochs and stopping criteria:
  Training continues until either:
  - The partial derivatives of the error function fall below the threshold (threshold = 0.1), or
  - The maximum number of steps (stepmax = 1 × 10⁹) is reached.

### 4.5 Regularization

- Early stopping, dropout, and weight decay are not natively supported in the base neuralnet package.
  Regularization was implicitly controlled through the threshold-based stopping criterion.
